# The Potassium Transporter Hak1 in *Candida Albicans,* Regulation and Physiological Effects at Limiting Potassium and under Acidic Conditions

**DOI:** 10.3390/jof7050362

**Published:** 2021-05-06

**Authors:** Francisco J. Ruiz-Castilla, Elisa Rodríguez-Castro, Carmen Michán, José Ramos

**Affiliations:** 1 Department of Agricultural Chemistry, Edaphology and Microbiology, University of Córdoba, E-14071 Córdoba, Spain; a32rucaf@uco.es (F.J.R.-C.); q72rocae@uco.es (E.R.-C.); 2 Department of Biochemistry and Molecular Biology, Campus de Excelencia Internacional Agroalimentario CeiA3, University of Córdoba, 14071 Córdoba, Spain; bb2midoc@uco.es

**Keywords:** *Candida albicans*, potassium transporters, Hak1, lithium, pH

## Abstract

The three families of yeast plasma membrane potassium influx transporters are represented in *Candida albicans*: Trk, Acu, and Hak proteins. Hak transporters work as K^+^-H^+^ symporters, and the genes coding for Hak proteins are transcriptionally activated under potassium limitation. This work shows that *C. albicans* mutant cells lacking *CaHAK1* display a severe growth impairment at limiting potassium concentrations under acidic conditions. This is the consequence of a defective capacity to transport K^+^, as indicated by potassium absorption experiments and by the kinetics parameters of Rb^+^ (K^+^) transport. Moreover, *hak1−* cells are more sensitive to the toxic cation lithium. All these phenotypes became much less robust or even disappeared at alkaline growth conditions. Finally, transcriptional studies demonstrate that the *hak1−* mutant, in comparison with *HAK1+* cells, activates the expression of the K^+^/Na^+^ ATPase coded by *CaACU1* in the presence of Na^+^ or in the absence of K^+^.

## 1. Introduction

Potassium is of pivotal importance in living cells. It is an essential macronutrient that fulfils critical functions related to enzyme activation, osmotic adjustment, turgor generation, cell expansion, regulation of membrane electric potential, or pH homeostasis [1]. In the case of yeast cells, potassium is taken up and accumulated against high concentration gradients with the help of processes that involve the movement of the cation mediated by different transport systems embedded in the plasma membrane [2,3]. Most of the yeast species are endowed with several plasma membrane potassium-specific transporters that can be grouped into three families: Trk (Transport of potassium (kalium)), Hak (High-affinity potassium (kalium)), or Acu (Alkali cation uptake) [2]. While the members of the Trk family are present in all yeasts, Hak and Acu transporters have been found only in some non-conventional yeast species [4,5,6,7]. Hak transporters have been widely associated with K^+^ transport across membranes. The presence of Hak proteins has been reported also in mycelial fungi, e.g., *Neurospora crassa* [8,9]. Among other organisms, homologous proteins are encoded by the KT/HAK/KUP family of genes in plants [10,11,12]. In *Escherichia coli*, Kup is a constitutive low-affinity uptake system that operates as K^+^:H^+^ symporter [13].

The first report of a Hak transporter in yeasts described the expression of a *KUP* homologue from the soil yeast *Schwanniomyces occidentalis.* The gene was cloned into a *Saccharomyces cerevisiae* mutant lacking its endogenous potassium transport systems and conferred the capacity to mediate high-affinity K^+^ transport that exhibited an enormous concentrative capacity [14]. Later, *HAK* genes were identified in other non-conventional yeasts, such as *Hansenula polymorpha* [7], *Debaryomyces hansenii* [6], *Pichia sorbitophila* [4], or *Candida albicans* [5]. Fungal *HAK* genes and some members of the KT/HAK/KUP family in plants are strongly induced by K^+^ starvation [6,11,14]. Such high-affinity K^+^ transporters are expected to be K^+^:H^+^ symporters, although our knowledge about their molecular mechanisms is far from complete [15,16,17].

*Candida albicans* is an important opportunistic yeast pathogen. It is present in most healthy persons but, under certain circumstances, can cause a wide range of diseases [18,19]. Potassium homeostasis in this organism has not been studied in detail and deserves much more attention due to the importance of the cation in cell physiology and, thus, in pathogenesis. *C. albicans* belongs to the group endowed with the three types of plasma membrane potassium transporters mentioned above [5]. CaTrk1 has been partly characterized and it was proposed to be the major potassium transporter in *C. albicans* [20,21]. Recently, potassium requirements and transport characteristics in wild-type *C. albicans* cells were characterized and transcriptional regulation of the genes coding for the transporters was studied [22]. In addition, CaTrk1, CaHak1, and CaAcu1 were heterologously expressed in a mutant *S. cerevisiae* strain lacking its own potassium uptake systems. All three transporters were able to provide cells with the ability to grow with low amounts of external potassium, and their capacity to transport potassium was demonstrated [5,22].

The main objective of this work was to identify the physiological roles of Hak1 transporter in *C. albicans*. For that purpose, we took advantage of the availability of a homozygous deletion strain carrying a disrupted version of *CaHAK1* (ORF 19.6249). That mutant was previously reported to show a defect in infectivity but no effect in morphogenesis or proliferation [23]. Here we have analyzed potassium homeostasis in the Hak defective strain, and our results show that CaHak1 is an important factor influencing growth and cation transport at acidic pHs. Furthermore, we show that lack of the *CaHAK1* gene increases transcription of *CaACU1*, but it does not affect *CaTRK1*. Finally, we demonstrate that CaHak1 transporter is involved in tolerance to toxic lithium at low pH.

## 2. Materials and Methods

### 2.1. Yeast Strains and Cultivation

SN250 *HAK1+* and SN250 *hak1−* strains were used in this study [23]. Cells were grown in YNB based medium (0.17% YNB or 0.17% YNB-F, Formedium^TM^, Norfolk, UK) supplemented with 2% glucose, 0.4% ammonium sulphate, and the auxotrophic supplements (60 mg/L l-Leucine, 40 mg/L l-Histidine and 40 mg/L l-Arginine). The pH of the media was usually adjusted at pH 5.8 with NH_4_OH. When a different pH was required, the medium was buffered at pH 3.0, 4.5, or 7.5 with citric acid or HEPES. Solid media were prepared by adding 2% (*w*/*v*) agar to the previously described recipes. Cells were routinely grown at 28 °C. To obtain normal K^+^ cells, yeasts were grown in YNB supplemented with 30 mM KCl. For K^+^ starvation experiments, normal K^+^ cells were washed with sterile water and resuspended in K^+^-free medium (YNB-F) without added potassium for 3 h.

### 2.2. Growth Assays

Potassium requirements were determined both in solid and liquid media and the required pH was adjusted as mentioned above. Drop tests were performed with fresh cells resuspended in sterile water and adjusted to the same 1.0 initial A_600_. Ten-fold serial dilutions were prepared, and 5 μL aliquots of each dilution were spotted on the appropriate plates containing several potassium concentrations. Plates were then incubated for 24–48 h at 28 °C.

Growth curves at different KCl or LiCl concentrations were assayed in tubes of 10 mL with 5 mL of YNB-F medium supplemented with the corresponding amounts of salts. Cultures were inoculated at A_600_ 0.05 and the absorbance at 600 nm was followed for 24–48 h using a Spectronic 20 (Bausch and Lomb, Bridgewater, NJ, USA). Specific growth rates were calculated from the potassium growth curves [24].

### 2.3. Cation Content and Transport

Changes in extracellular potassium and the characteristics of Rb^+^ uptake were studied at several pHs. For pH 3.0 and pH 4.5 experiments, TRIS (10 mM) buffer was supplemented with 0.1 mM MgCl_2_, 0.1 mM CaCl_2_, and adjusted at pH 3.0 or 4.5, respectively, with citric acid. For pH 5.8 or 7.5 experiments, MES (10 mM) or HEPES (10 mM) buffer was supplemented with 0.1 mM MgCl_2_ and adjusted at the required pH with Ca(OH)_2_. In all cases, the buffers were supplemented with 2% glucose.

Variation of external potassium was determined after transferring K^+^-starved cells (A_600_ ≈ 0.5) to the corresponding uptake buffer. For these experiments, the buffers were supplemented with 25–28 μM K^+^. To determine the time course of the uptake, K^+^ changes were followed by taking samples at different times. The samples were then immediately filtered (Millipore, AAWP 0.8 μm pore size, Darmstadt, Germany) to separate cells, and potassium levels were measured in the remaining liquid fraction by atomic absorption spectrometry.

To study the characteristics of Rb^+^ uptake, normal K^+^ cells or K^+^-starved cells were washed and suspended (A_600_ ≈ 0.3) in uptake buffers prepared as previously described. Eight to twelve concentrations of RbCl ranging from 20 μM to 100 mM were used to deduce the kinetic parameters of transport. The corresponding amount of RbCl was added to the buffer at time zero and aliquots were withdrawn at various times. To estimate intracellular alkali-metal-cation content, cell samples were collected from liquid media or buffers on Millipore filters (0.8 μm pore size) and rapidly washed with 20 mM MgCl_2_. The cells were then treated with 0.2 M HCl and 10 mM MgCl_2_ and the extracts were analyzed by atomic emission spectrophotometry [25].

Extracellular potassium is expressed in micromolar units. All intracellular cation values are expressed as nmols of cation per mg dry weight of cells [26].

### 2.4. RNA Isolation and Reverse Transcription

Total RNA from SN250 *HAK1+* and SN250 *hak1−* strains was extracted using the TRI-REAGENT reactive (Sigma-Aldrich, Darmstadt, Germany). Cells grown or incubated under different conditions (cells starved for potassium in YNB-F medium, cells grown in YNB medium with 1 M NaCl, cells grown in YNB medium adjusted at pH 4.5, 5.8, or 7.5 as described above) were collected, washed with sterile cold water, and resuspended in 1 mL of TRI-REAGENT plus approximately 200 μL 0.5-mm glass beads. For disruption, yeasts were vortexed 10 times for 1 min with intervals of at least 1 min on ice, incubated 5 min at 70 °C, and followed by another 10 times of 1 min vortexing with cooling intervals. Afterward, the standard TRI-REAGENT protocol for RNA isolation was followed. Isolated RNA samples were treated using DNase I (New England Biolabs, Ipswich, MA, USA) to remove contaminating DNA until no PCR amplification was observed without prior cDNA synthesis. RNA sample quality and quantification were performed spectrophotometrically. At least two RNA preparations were isolated for each experimental condition. A total of 1 μg from each RNA sample was retrotranscribed with Kit iScript™ cDNA Synthesis Kit (Bio-Rad, Hercules, CA, USA) on three separate occasions that were pooled together before PCR amplification.

### 2.5. Real-Time PCR

The primers sequences used for the amplification of *TRK1*, *ACU1,* and *HAK1* genes were described and their quality was tested in a previous published article [22].

The PCR amplification was carried out in a mixture (25 μL final volume) with IQ^TM^ SYBR^®^ Green Supermix (Bio-Rad, Hercules, CA, USA), 1 μL of cDNA, plus 0.1 μM of the specific primers. PCR reactions were performed at least in triplicate. Real-time PCR conditions were an initial denaturation step, 95 °C for 3 min, followed by forty PCR cycles consisting of 15 s of denaturation at 95 °C, and 30 s of annealing plus elongation at 70 °C. Finally, melting curves were determined, and no primer dimers were detected.

### 2.6. Statistics

All experiments were repeated at least three times and three technical replicates for each sample were performed. Data were analyzed with Microsoft Excel 2016 software and the significance of differences between mean values was determined by GraphPad Prism 7. Significant differences are indicated with asterisks (* *p <* 0.05, ** *p <* 0.01, *** *p <* 0.001).

## 3. Results and Discussion

**Figure 1:**
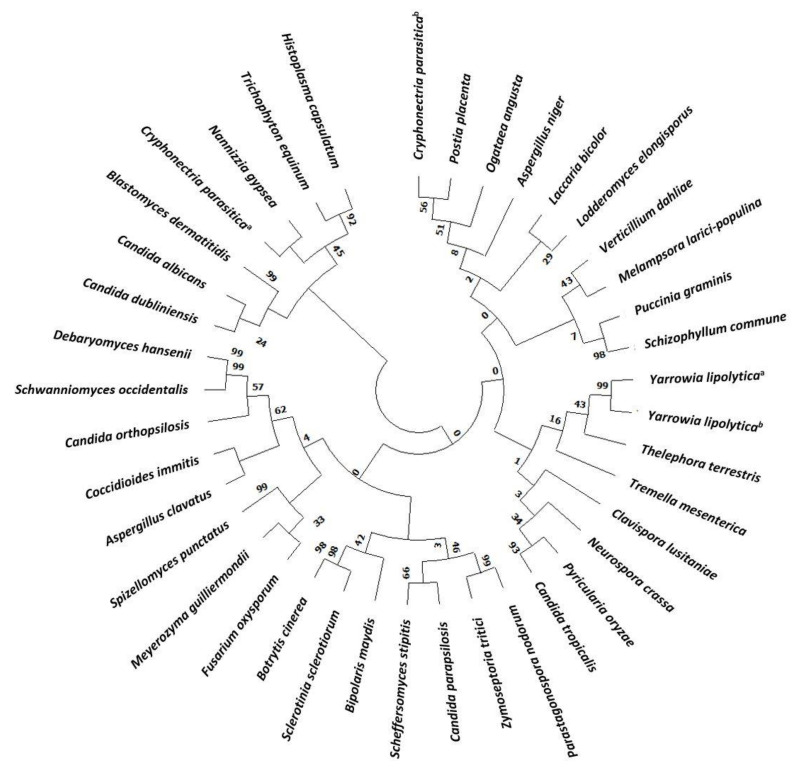
Phylogenetic tree of Hak family proteins in main fungi. Databases used for identification were: National Center for Biotechnology Information (NCBI) and Joint Genome Institute (JGI). Evolutionary relationships were assessed using the MEGA X software (https://www.megasoftware.net, accessed on 5 March 2021). Parameters were preserved by default. Phylogenetic tree was constructed with the Neighbor joining likelihood approach. The Bootstrap consensus tree was inferred from 500 sampling replicates. Protein sequences with more than 30% homology to CaHAK1 and that putative belong to the KT/HAK/KUP family were selected. Protein IDs are included in Supplementary Table S1 (TS1).

The evolution of Hak transporters is a very interesting topic since they are present in many different organisms, widely distributed along the evolutionary scale [27]. A phylogenetic tree of Hak in main Fungi has been constructed (Figure 1 and Table S1). About one third of the *HAK* genes identified belong to yeast and most of them are *Candida* species. Despite being present in many fungi, many others have lost their corresponding genes during evolutionary history and, in fact, neither the budding yeast *Saccharomyces cerevisiae* nor the fission yeast *Schizosaccharomyces pombe* carries genes belonging to the family [2]. Hak transporters are members of the amino acid-polyamine organocation (APC) superfamily. Although the molecular bases for transport in the potassium importer KimA from *Bacillus subtilis* have been recently unravelled [28], the detailed structural information of KT/HAK/KUP transport mechanisms remains largely unknown.

### 3.1. Potassium Requirements and Transport Characteristics in HAK1+ and hak1− Candida albicans

**Figure 2:**
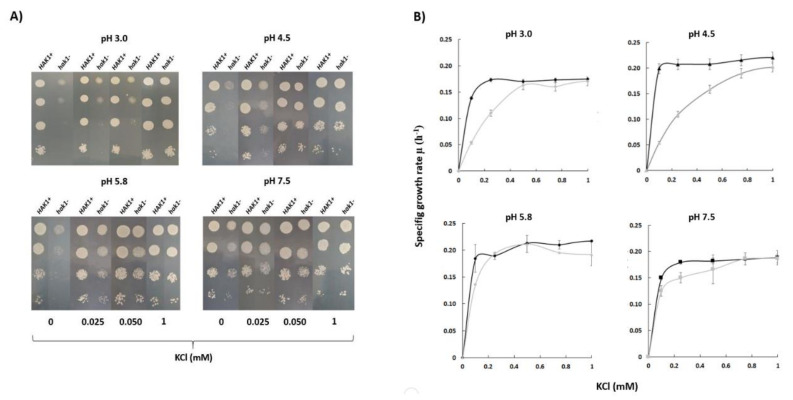
Potassium requirements for growth at different pHs in *C. albicans HAK1+* and *hak1−* strain. (**A**) Serial dilutions of yeast suspensions were inoculated on YNB-F plates containing the indicated concentrations of KCl and adjusted at the required pH as described in the Materials and Methods. The pictures were taken after 48 h incubation at 28 °C. Drop tests were repeated three times and a representative experiment is shown. (**B**) Specific growth rate of *C. albicans HAK1+* (Black) and *hak1−* (Gray) at different K^+^ concentrations in liquid YNB-F medium supplemented with the indicated amounts of KCl and adjusted at the required pH as described in the Materials and Methods. Cultures were inoculated at A_600_ 0.05, growth was monitored during 48 h, and specific growth rates were deduced from data in growth curves. Mean values ± standard deviation obtained in three independent experiments are plotted.

Experiments in YNB-F solid media showed that *Candida albicans* SN250 *HAK1+* strain was able to grow with low potassium concentrations at a wide range of pHs (Figure 2A). On the other hand, growth of the corresponding *hak1*− strain was severely affected when both the potassium concentration and pH in the medium were low (Figure 2A). The differences between both strains were more notable at pH 3.0, where the strain with the complete potassium transporter Hak1 (*HAK1+*) showed a much better growth than the strain with the disrupted gene. Nevertheless, these differences decreased as the pH increased and were almost non-existent at pH 7.5 (Figure 2A).

To quantify in more detail the potassium requirements of both strains, growth experiments in YNB-F liquid medium supplemented with several KCl concentrations were also performed and specific growth rates were calculated (Figure 2B). The results obtained showed a similar trend with those obtained in the solid medium. Under conditions of pH 7.5 or 5.8, no great differences between the *HAK1+* and *hak1−* strains were observed. On the other hand, the behaviour of both strains was different when the pH of the media was decreased. Under these conditions, the *hak1−* strain showed lower specific growth rates at limiting potassium concentrations compared to the *HAK1+* (Figure 2B), which is consistent with results in Figure 2A. These results would agree with a previous work describing that, in *Schwanniomyces occidentalis*, the Hak1 transporter is functional at low pH but fails at high pH, and that the Trk1 transporter functions at neutral and high pH but shows a more defective work at low pH [29].

**Figure 3:**
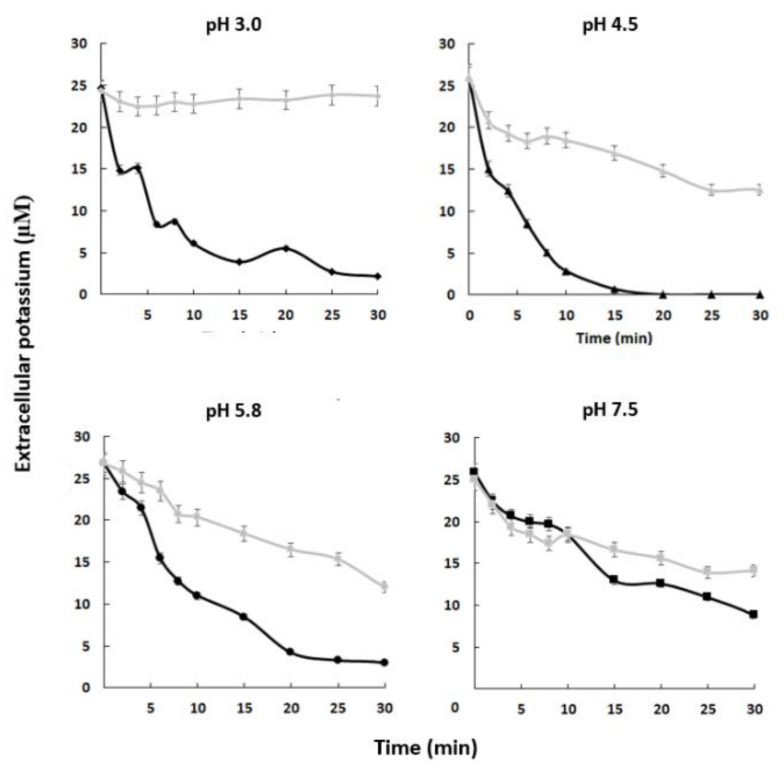
Absorption of external potassium in *C. albicans HAK1+* and *hak1−* strains. K^+^-starved cells were resuspended (A_600_ 0.5) in uptake buffer adjusted at the required pH (see the Materials and Methods) and containing 25–28 µM K^+^ approx. Samples of *HAK1+* (black) and *hak1−* (gray) cells were taken at the indicated times, filtered, and K^+^ in the medium was measured. Mean values ± standard deviation obtained in three independent experiments are plotted.

Our results suggest that the *C. albicans* strain containing the complete *HAK1* gene has a greater capacity to absorb potassium from the external environment than the *hak1* defective one and that this is especially relevant at low concentrations of the cation and at acidic pHs. To confirm this idea, potassium-starved *HAK1+* and *hak1−* cells were suspended in uptake buffers adjusted at different pHs containing micromolar concentration of potassium. Then, changes in the extracellular potassium levels were determined. Results obtained from these experiments are shown in Figure 3. The extracellular potassium levels showed a sharp decrease when cells of the *HAK1+* strain were present, suggesting an immediate uptake from the medium under the conditions studied. On the other hand, the *hak1−* strain showed some difficulties in taking up potassium, as the extracellular levels showed a smoother descent or even no changes, particularly at low environmental pHs. For example, after ten minutes of incubation at pH 3.0, the differences observed between the strains were remarkable, showing that the *HAK1+* strain absorbed almost all the potassium from the extracellular medium while the *hak1−* strain was not able to decrease external potassium. However, these differences decreased when the pH increased. This can be observed in the results obtained at pH 7.5, where after ten minutes of incubation both strains had a similar behaviour. These results confirm the relevance of the potassium transporter Hak1 for *C. albicans* cells to take up extracellular potassium in acidic environments.

To further characterise potassium uptake, we performed a detailed analysis of the kinetics of Rb^+^ uptake (a commonly used K^+^ transport analogue) in cells grown under normal K^+^ conditions and in K^+^-starved cells at the different pHs previously tested (Table 1). We found minor differences in the apparent kinetic constants for rubidium transport between strains in normal potassium cells indicating that under these conditions the relevance of Hak1 transporter is not very important. However, when the kinetics of Rb^+^ transport were studied in potassium-starved cells, we found significant differences, specially at low pH. It should be noted that as the pH decreased, the differences between the strains became more evident both in their V_max_ and in their K_m_. For example, the estimated kinetic constants for rubidium transport at pH 3.0 showed great differences between the strains. Specifically, in the *HAK1+* strain vs. the *hak1−*, the V_max_ of rubidium transport was 12.9-fold higher and the Km for the cation was 41.6-fold lower. However, these differences changed when pH increased and almost disappeared at pH 7.5 (V_max_ was only 1.3-fold higher and K_m_ 2.6-fold lower in the strain with *HAK1* complete vs. the defective mutant).

All together these results suggest that the expression of *HAK1* gene or the activity of the corresponding protein is much higher in potassium-starved cells and, once again, point to the fact that the extracellular proton levels may play a role in the transport process.

The results shown above are in agreement with the hypothesis of a potassium:proton symporter working with high affinity for the cation as previously proposed in *Schwanniomyces occidentalis* [2,29,30]. At this point it is important to highlight that it has been proposed that, in addition to their cation transport activities, some potassium transporters can influence other important processes. In fact, several studies carried out in *Arabidopsis* have shown that members of the HAK/KUP/KT family are involved in the regulation of cell size, auxin distribution, or osmotic stress adaptation [31,32]. Moreover, in the case of *C. albicans*, the *hak1−* mutant was isolated and partially characterized, not because of its potassium transport characteristics, but on the basis of a defective infectivity [23].

### 3.2. Transcriptional Regulation of the C. albicans Plasma Membrane Potassium Transporters

As mentioned throughout this work, three types of plasma membrane potassium transporters have been identified in *C*. *albicans*: Trk1, Hak1, and Acu1 [5]. The two strains used in this work are derivatives of *C. albicans* SC5314, widely used as a wild-type reference strain. Regarding potassium transporters, SC5314 has an amber stop codon mutation inside *ACU1* coding region and, thus, should lack viable Acu1 proteins although *ACU1* transcript levels could be determined in that strain [5,22,33].

**Figure 4:**
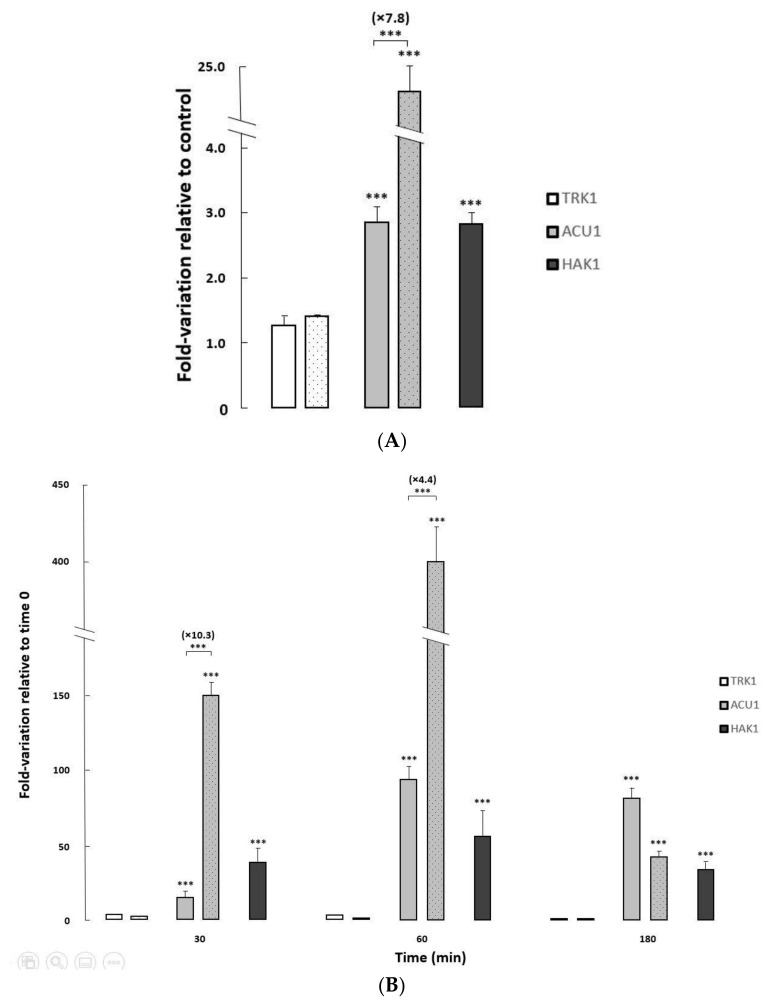
Changes in expression levels of potassium transporter genes in *C*. *albicans HAK1+* and *hak1−* strains. (**A**) The cells were grown overnight in YNB liquid medium (Control) and in YNB medium supplemented with 1 M NaCl to an A_600_ of 0.6–0.7. (**B**) Normal-K^+^ cells were grown to exponential phase, washed and resuspended in YNB-F without KCl, and further incubated. Samples were taken at different times during potassium starvation (0, 30, 60 and 180 min). All transcript levels are referred to those for the controls (**A**) or time 0 (**B**). The *HAK1+* strain was represented by filled bars while the mutant strain was represented by dotted bars. Mean values ± standard deviations were obtained from at least three independent experiments. Statistically significant compared to the controls/time 0 and comparing the strains (square brackets) expressed as: *** *p <* 0.001.

To further investigate the regulation of the three transport mechanisms at transcriptional level, and the impact of *HAK1* deletion on that regulation, the expression of their corresponding genes was studied in the isogenic *HAK1+* and *hak1−* strains used in this work, under different growth conditions (Figure 4).

Changes in the pH of the media only provoked very slight variations on the transcription of *TRK1*, *ACU1,* and *HAK1* genes in both strains studied (Elisa Rodríguez-Castro and José Ramos, Final Bachelor’s degree thesis, under progress). On the other hand, exposure to saline stress or potassium-limiting conditions triggered major changes in *ACU1* and *HAK1* transcription levels, particularly in the former where huge increments (>400-fold) could be observed upon potassium starvation (Figure 4).

Both *ACU1* and *HAK1* mildly increased their mRNA when the control *HAK1+* strain was grown on media supplemented with sodium chloride. Furthermore, salt-induced *ACU1* transcription was further fueled in cells lacking the Hak1 transporter (Figure 4A), suggesting a compensation mechanism.

To study the effect of potassium deprivation, cells were grown to exponential phase in regular media, then changed to limiting potassium conditions, and the transcriptional levels of the three potassium transporter genes were followed at different times after the change (Figure 4B). In the *HAK1+* control strain, and in concordance with a previous work [22], *ACU1* and *HAK1* genes increased their expression upon potassium starvation with a peak at 60 min after the deprivation, although the induction was larger for *ACU1*. As observed for the saline stress response, induction of *ACU1* transcription upon potassium deprivation stress was further increased (up to 10.3-fold) in the *hak1−* strain compared to the *HAK1*+, additionally supporting that the lack of Hak1 could affect the level of *ACU1* transcripts.

It is usually reported that *TRK* genes are not significantly regulated at transcriptional level [2,3]. In our case, *CaTRK1* mRNA presented very moderate changes upon saline stress or upon potassium deprivation (Figure 4).

### 3.3. Role of Hak1 in Tolerance to the Toxic Lithium Cation

Lithium is a toxic alkali cation that uses potassium transporters in the yeast plasma membrane to enter the cell. Therefore, it is conceivable that *C. albicans* transporters may play a role in salt tolerance, most likely by interfering with the accumulation of toxic cations. For this reason, we tested the tolerance of our *HAK1*+ and *hak1−* strains to this toxic cation in liquid media adjusted at different pHs and supplemented with several LiCl concentrations.

**Figure 5:**
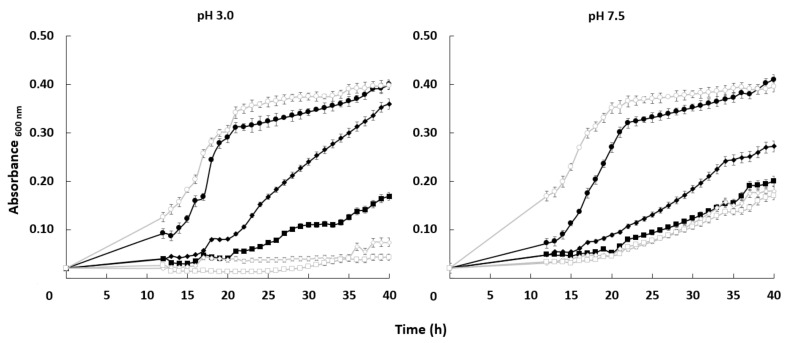
Effect of LiCl on the growth of *C. albicans HAK1+* and *hak1−* strains at different pHs. Cells were grown in liquid YNB medium supplemented with the indicated amounts of LiCl and adjusted at pH 3.0 or 7.5 as described in the Materials and Methods. The concentrations of LiCl studied were: 0 (circle), 0.15 (diamond) or 0.30 (square) M LiCl. Cultures were inoculated at A_600_ 0.05 and growth of *HAK1+* (filled symbols) and *hak1−* (open symbols) strains was monitored during 40 h. Mean values ± standard deviation obtained in three independent experiments are plotted.

The growth curve of both strains was very similar in the absence of the toxic cation. When lithium chloride was added to the medium, the growth of both strains was affected but the mutant was more sensitive under some specific conditions. Differences between the *HAK1+* strain and the one with the disrupted gene were more significant at pH 3.0. At acidic conditions, 0.15 M LiCl impaired growth of the mutant much more strongly than in the case of *HAK1+* cells. At pH 7.5, the differences in tolerance between strains were much less important (Figure 5).

To obtain further information on the reasons behind the lithium tolerance due to the activity of Hak1 transporter, we measured intracellular lithium and potassium content in cells of both strains grown under the same conditions used for the lithium toxicity growth assays (Table 2). Lithium and sodium toxicity in yeasts has been usually linked to a higher intracellular accumulation of the toxic cation and, at the same time, to lower concentrations of internal potassium [2,34]. We did not find differences in intracellular lithium content between strains under any condition studied. However, the *HAK1+* strain was able to keep moderately higher amounts of potassium than the mutant when grown at pH 3.0, both in the presence of 0.15 or 0.30 M LiCl. These differences disappeared when the experiment was performed at pH 7.5 (Table 2 and Elisa Rodríguez-Castro and José Ramos, Final Bachelor’s degree thesis, under progress). Our results indicate that the role of the CaHak1 transporter in lithium tolerance can be indirect since it does not affect the accumulation of the toxic cation, but it helps to keep an appropriate potassium homeostasis [22].

In conclusion, we show in this research that Hak1 is a crucial potassium transporter in *C. albicans*. This transporter is fully required to provide potassium for growth under some specific conditions such as low external potassium and acidic pHs. Furthermore, Hak1 contributes to avoid lithium toxicity at low pH. Further studies are needed to determine the overlapping functions of the different potassium transporters under variable environmental conditions.

## Figures and Tables

**Table 1:** Apparent kinetic parameters for rubidium transport in normal K^+^ cells and K^+^-starved cells of *Candida albicans* strains.

pH	*HAK1*	Normal K^+^ Cells		K^+^ -Starved Cells	
		V_max_	K_m_	V_max_	K_m_
		(nmol∙mg^−1^∙min^−1^)	(mM)	(nmol∙mg^−1^∙min^−1^)	(mM)
3.0	+	0.63 ± 0.20	1.63 ± 0.55	11.49 ± 0.20	0.09 ± 0.02
	−	0.45 ± 0.22	1.88 ± 0.42	0.89 ± 0.07 ***	3.75 ± 0.05 ***
4.5	+	0.91 ± 0.12	0.95 ± 0.04	13.71 ± 0.58	0.03 ± 0.02
	−	0.74 ± 0.09	0.87 ± 0.05	3.69 ± 0.43 ***	0.59 ± 0.06 ***
5.8	+	1.96 ± 0.33	0.25 ± 0.02	9.79 ± 0.43	0.09 ± 0.02
	−	1.34 ± 0.20 *	0.32 ± 0.03	5.57 ± 1.25 **	0.55 ± 0.17 **
7.5	+	2.68 ± 0.27	0.36 ± 0.03	9.92 ± 1.65	0.28 ± 0.08
	−	2.01 ± 0.11 *	0.40 ± 0.07	7.89 ± 1.79	0.74 ± 0.25 **

Kinetic parameters of rubidium transport were calculated after plotting data using GraphPad Prism v7. Mean values ± standard deviations were obtained from three independent experiments. Statistically significant data were expressed as: * *p <* 0.05, ** *p <* 0.01, *** *p <* 0.001.

**Table 2:** Intracellular lithium and potassium in *Candida albicans* strains.

pH	*HAK1*	0.30 M LiCl	
		Lithium (nmol∙mg^−1^)	Potassium (nmol∙mg^−1^)
3.0	+	13.9 ± 0.4	242.7 ± 12.5
	−	14.8 ± 0.5	185.9 ± 10.4 **
7.5	+	13.1 ± 3.8	126.3 ± 12.8
	−	14.3 ± 4.6	122.3 ± 7.3

Cells were grown in YNB medium adjusted at pH 3.0 or 7.5 and supplemented with 0.15 or 0.30 M LiCl. Mean values ± standard deviations were obtained from three independent experiments. Statistically significant data were expressed as: ** *p <* 0.01.

## Data Availability

Data is contained within the article.

## Supplementary Materials

The following are available online at https://www.mdpi.com/article/10.3390/jof7050362/s1. Table S1: Proteins included in the Phylogenetic tree of Hak family in Fungi.
