# Supplementary material for: The Potassium Transporter Hak1 in Candida Albicans, Regulation and Physiological Effects at Limiting Potassium and under Acidic Conditions
Source: J Fungi (Basel). 2021 May 6;7(5):362. doi: 10.3390/jof7050362 (PMC8148600; doi:10.3390/jof7050362)
Supplement: Supplementary file 1 [file jof-07-00362-s001.zip › jof-1177578-supplementary.pdf]

Table S1. Proteins included in the Phylogenetic tree of Hak family in Fungi

ID sequence	Fungi Strains
XP_016607719.1	*Spizellomyces punctatus* DAOM BR117
ABI37006.2	*Debaryomyces hansenii* CBS 767
XP_718838.2	*Candida albicans* SC5314
XP_001383251.2	*Scheffersomyces stipitis* CBS 6054
XP_001528091.1	*Lodderomyces elongisporus*
VBB78842.1	*Yarrowia lipolytica*[a]
XP_501676.1	*Yarrowia lipolytica*[b]
XP_002417280.1	*Candida dubliniensis* CD36
XP_036666213.1	*Candida parapsilosis*
EDK41390.2	*Meyerozyma guilliermondii*
OVF10943.1	*Clavispora lusitaniae*
XP_002549931.1	*Candida tropicalis* MYA-3404
XP_003866449.1	*Candida orthopsilosis* Co 90-125
AAB17122.2	*Schwanniomyces occidentalis*
CCE34941.1	*Ogataea angusta*
XP_007007047.1	*Tremella mesenterica* DSM 1558
XP_001876329.1	*Laccaria bicolor* S238N-H82
XP_003032151.1	*Schizophyllum commune* H4-8
XP_007407239.1	*Melampsora larici-populina* 98AG31
KAF9808492.1	*Postia placenta* MAD-698-R-SB12
XP_003325997.2	*Puccinia graminis f. sp. tritici* CRL 75-36-700-3
KAG5294344.1	*Histoplasma capsulatum* G217B
EEQ88319.2	*Blastomyces dermatitidis* ER-3
XP_001247463.2	*Coccidioides immitis* RS
XP_003169811.1	*Nannizzia gypsea* CBS 118893

EGE03791.1	*Trichophyton equinum* CBS 127.97
SMY29655.1	*Zymoseptoria tritici* ST99CH_1A5
XP_001273808.1	*Aspergillus clavatus* NRRL 1
XP_001399699.2	*Aspergillus niger* CBS 513.88
XP_014080355.1	*Bipolaris maydis* ATCC 48331
XP_001804449.1	*Parastagonospora nodorum* SN15
XP_964946.1	*Neurospora crassa* OR74A
XP_040773768.1	*Cryphonectria parasitica* EP155[a]
KAF3770430.1	*Cryphonectria parasitica* EP155[b]
ELQ63511.1	*Pyricularia oryzae* 70-15
XP_009655039.1	*Verticillium dahliae* VdLs.17
XP_018242043.1	*Fusarium oxysporum*
XP_001554184.2	*Botrytis cinerea* B05.10
XP_001591952.1	*Sclerotinia sclerotiorum* 1980 UF-70
KAF9784960.1	*Thelephora terrestris*

[a, b] refer to different Haks in the same species
